# WT1基因定量动态监测预测伴MLL基因重排急性髓系白血病造血干细胞移植后复发的临床价值

**DOI:** 10.3760/cma.j.issn.0253-2727.2022.09.013

**Published:** 2022-09

**Authors:** 新鑫 刘, 军 孔, 信安 潘, 竞 刘, 亚溱 秦, 英军 常, 昱 王, 晓辉 张, 兰平 许, 晓军 黄, 晓甦 赵

**Affiliations:** 北京大学人民医院血液科、北京大学血液病研究所、国家血液系统疾病临床医学研究中心、造血干细胞移植治疗血液病北京市重点实验室，北京 100044 Peking University People's Hospital, Peking University Institute of Hematology, National Clinical Research Center for Hematologic Disease, Beijing Key Laboratory of Hematopoietic Stem Cell Transplantation, Beijing 100044, China

对于伴混合系白血病基因重排（Mixed Lineage leukemia rearrangement, MLL-r）的AML，MLL相关的融合基因可以作为有效的微小残留病（MRD）标志，对造血干细胞移植后的复发进行有效预测[1]。伴不同MLL-r的AML初诊时融合基因定量水平不同，随着肿瘤负荷变化的动力学也不尽相同，因此，可能并非所有类型的MLL-r都是最佳的分子MRD监测指标。此外，少数患者会出现克隆演变，流式细胞术（FCM）检测AML MRD的敏感性（10^−3^～10^−4^）一般低于分子生物学检测（10^−4^～10^−5^）[2]，而WT1作为泛白血病标志物可用于对大多数AML的MRD进行监测[3]。临床中我们会同时监测FCM、MLL相关融合基因以及WT1水平，观察伴有MLL-r的AML的MRD状态，预警移植后白血病复发。本研究拟比较WT1和MLL-r作为伴MLL-r的AML的MRD标志，预测移植后复发的阳性预测值和阴性预测值，以评估其临床应用价值。

## 病例与方法

1. 病例：2008年7月至2020年10月期间于北京大学人民医院血液科确诊的AML伴MLL-r并接受造血干细胞移植的连续患者100例，患者的诊断依据MICM标准[4]。纳入标准：①年龄>12岁；②原发性AML；③移植后MRD监测同时检测了MLL-r和WT1。排除标准：①继发性AML或者治疗相关性AML；②临床随访信息不全。

2. 方法：所有患者均进行了形态学、常规细胞遗传学、免疫表型分析，具体操作步骤参考文献[5]。应用实时定量PCR（RQ-PCR）方法检测12种MLL融合基因（MLL-AF4、MLL-AF6、MLL-AF9、MLL-AF10、MLL-ELL、MLL-ENL、MLL-AF1p、MLL-AF1q、MLL-SETP6、MLL-SETP9、MLL-AF17和MLL-AFX），具体方法参照文献[6]。WT1基因表达水平（％）＝WT1拷贝数/ABL拷贝数×100％，WT1>0.6％定义为阳性[7]–[8]。MLL-r>0％定义为阳性[1]。

3. 移植方案：对于HLA不完全相合的患者接受以下治疗：甲环亚硝脲（Me-CCNU）250 mg/m^2^，−3 d，1次；白消安（BU）3.2 mg·kg^−1^·d^−1^静脉滴注，−8～−6 d；阿糖胞苷（Ara-C）4 g·m^−2^·d^−1^，−10～−9 d；兔抗胸腺细胞球蛋白（ATG）2.5 mg·kg^−1^·d^−1^，−5～−2 d；环磷酰胺（CTX）1.8 g·m^−2^·d^−1^，−5～−4 d。对于HLA相合的同胞供者移植（MSDT），Ara-C减量为2 g·m^−2^·d^−1^并且加用羟基脲（80 mg/kg 总剂量）。所有患者均接受了包括G-CSF（5 µg·kg^−1^·d^−1^，5 d）动员的骨髓和外周血的同种异基因移植混合物。未处理的外周血干细胞（在G-CSF后第5天收集）和骨髓（在G-CSF后第4天收集）在收集当天回输。采用霉酚酸酯、甲氨蝶呤和环孢素A预防移植物抗宿主病[9]。

4. 复发干预措施：对移植后MRD阳性患者进行的干预措施包括减停免疫抑制剂、使用干扰素、联合化疗的改良供者淋巴细胞输注等。如患者发生严重的移植物抗宿主病、感染或器官衰竭等，暂不进行复发干预[10]。

5. 临床评估：移植后骨髓或髓外复发标准参考文献[3]。

6. 随访：通过查阅患者电子病历进行随访，随访截止日期为2021年9月30日，中位随访时间为1 192（49～4 647）d。

## 结果

1. 患者一般临床特征：100例伴有MLL-r融合基因的AML患者根据MLL-r的种类分为四组：MLL-AF9（35例），MLL-AF10（19例），MLL-AF6（27例）和其他MLL-r（19例，其中1例AF17，2例AF1q，2例AF4，11例MLL-ELL，1例MLL-ENL，2例MLL-SEPT9）。四组患者的临床资料见表1。各组伴MLL-r的AML患者主要分布在M_4_、M_5_两个亚型中。

**表1:** 100例伴MLL基因重排的急性髓系白血病造血干细胞移植患者的临床特征

临床特征	MLL-AF9（35例）	MLL-AF10（19例）	MLL-AF6（27例）	其他MLL-r（19例）
年龄［岁，*M*（范围）］	35（12~59）	25（12~54）	28（17~62）	36（14~50）
FAB分型（例）				
M_0_	0	0	0	0
M_1_	0	0	0	1
M_2_	4	2	1	6
M_4_	1	1	9	9
M_5_	30	16	16	3
未分类	0	0	1	0
患者性别（例，男/女）	20/15	8/11	17/10	8/11
供者性别（例，男/女）	23/12	13/6	17/10	13/6
移植类型（例）				
HLA相合	10	8	6	3
HLA不完全相合	25	11	21	16

2. 移植后WT1和MLL-r动态变化：对100例接受造血干细胞移植的患者移植后的WT1和MLL-r的动态变化进行分析，结果见表2。MLL-AF9组，WT1预测复发的阳性和阴性预测值分别为53％和95％。26例患者的MLL-r持续阴性，未观察到复发。MLL-r预测复发的阳性和阴性预测值分别为100％和100％。7例WT1转阳（0.72％～1.4％）并且MLL-r阴性的患者，未出现复发。

**表2:** 移植后患者WT1和MLL-r的动态变化情况

组别	MLL-r阳性					WT1阳性				
	例数	复发（例）	距离复发时间［d, *M*（范围）］	阳性预测值（％）	阴性预测值（％）	例数	复发（例）	距离复发时间［d, *M*（范围）］	阳性预测值（％）	阴性预测值（％）
MLL-AF9	9	9	18（0~259）	100	100	15	8	35（0~1361）	53	95
MLL-AF10	6	5	0（0~556）	83	92	6	3	146（143~556）	50	77
MLL-AF6	18	17	133（15~698）	94	100	20	16	76（0~1559）	80	86
其他MLL-r	7	5	72（11~724）	71	100	8	5	168（31~596）	63	100

MLL-AF10组，WT1预测复发的阳性和阴性预测值分别为50％和77％。13例患者MLL-r阴性，1例发生髓外复发，12例未复发。MLL-r预测复发的阳性和阴性预测值分别为83％和92％。2例WT1升高（0.75％～1.8％）并且MLL-r阴性，未复发。

MLL-AF6组，WT1预测复发的阳性和阴性预测值分别为80％和86％。9例患者的MLL-r持续阴性，且均未复发。MLL-r预测复发的阳性和阴性预测值分别为94％和100％。4例WT1升高（0.8％～2％）并且MLL-r阴性，未复发。

其他MLL-r组，WT1预测复发的阳性和阴性预测值分别为63％和100％。12例患者的MLL-r持续阴性，未观察到复发。MLL-r预测复发的阳性和阴性预测值分别为71％和100％。1例WT1升高（0.67％）并且MLL-r阴性，未复发。

对于100例整体伴MLL-r的AML患者，WT1预测复发的阳性和阴性预测值分别为65％和90％；MLL-r预测复发的阳性和阴性预测值分别90％和98％。四组患者移植后从WT1表达异常升高到复发的中位时间和MLL-r从转阳到复发的中位时间见表2。对同时间点的FCM分析显示：MLL-AF9组9例FCM^+^患者均复发，距复发中位时间为0（0～50）d；MLL-AF10组中6例FCM^+^患者中有4例复发，距复发中位时间为0（0～143）d；MLL-AF6组17例FCM^+^患者均复发，距复发中位时间为33（0～472）d；其他MLL-r组6例FCM^+^患者中有5例复发，距复发中位时间为40（0～692）d。

3. 造血干细胞移植后复发患者中WT1^+^和MLL-r^+^情况分析：造血干细胞移植后复发患者中WT1^+^和MLL-r^+^的时间点见表3。大部分患者的MLL-r转阳时间点早于或与WT1同时转阳［早于或同时转阳比例，MLL-AF9：88.9％（8/9），MLL-AF10：66.7％（4/6），MLL-AF6：76.5％（13/17），其他MLL-r：80.0％（4/5）］。

**表3:** 移植后复发患者中MLL-r^+^者WT1转阳情况（例）

复发患者中MLL-r^+^	例数	MLL-r^+^早于WT1^+^	MLL-r和WT1同时转阳	MLL-r^+^在WT1^+^之后
MLL-AF9	9	2	6	1
MLL-AF10	6	2	2	2
MLL-AF6	17	11	2	4
其他MLL-r	5	3	1	1

4. 移植后MRD阳性患者的复发干预：MLL-AF9组9例移植后MRD阳性（MLL-r转阳）患者接受化疗、DLI或者干扰素治疗，其中7例MRD未转阴，后复发死亡；2例MRD转阴后存活。MLL-AF10组6例移植后MRD阳性患者接受干预治疗，其中4例未转阴，后复发死亡；2例转阴后存活。MLL-AF6组11例移植后MRD阳性患者接受干预治疗，皆未转阴，后复发死亡。对于其他MLL-r组，3例移植后MRD阳性患者接受干预治疗，皆未转阴，后复发死亡。

## 讨论

根据欧洲白血病网络小组（ELN）的危险分层，伴MLL融合基因重排的AML患者属于中高危人群[11]。伴MLL-r的AML患者因预后相对不良，符合条件的患者都应该建议进行HSCT，但是移植后复发的问题值得关注。目前MLL-r和WT1相比，哪个更适合作为MRD监测指标尚无定论，有必要对WT1作为对伴MLL-r的移植后AML的MRD标志的临床价值进行进一步探究。我们通过回顾分析得到了WT1在四种不同类型MLL-r中预测移植后复发的阳性预测值和阴性预测值，并将其与MLL-r的阳性预测值和阴性预测值进行了比较，WT1预测移植后复发的阳性预测值和阴性预测值均低于MLL-r，表明WT1在HSCT后MLL融合基因阳性AML MRD监测中的作用不够显著。

随后我们对allo-HSCT复发的患者WT1转阳与MLL-r转阳的时间点进行了分析，发现大部分患者的MLL-r转阳时间点早于或与WT1同时转阳。从MRD转阳时间角度上看，WT1监测伴MLL-r的AML患者移植后MRD的作用并不优于MLL-r。研究显示WT1预测AML移植后复发的特异性并非100％，并且WT1在预测AML移植后复发时的阈值也并不完全相同[1],[12]–[13]。我们的研究结果表明部分单纯WT1移植后轻度或一过性升高的患者，融合基因阴性，也没有复发，提示WT1不够特异，可能有其他因素影响其表达。所以移植后如果单纯WT1轻度升高，而不伴MLL-r转阳，临床上可以观察，综合判断，避免过度干预带来的不良预后。

本研究入组病例较少，且为单中心、回顾性分析。期望未来有多中心、前瞻性临床试验针对此问题进一步探究，并能继续明确各种类型MLL-r融合基因随治疗的动力学变化，及与其他MRD指标的相关性。

综上，伴有MLL-r的AML中，WT1预测复发的阳性预测值和阴性预测值低于MLL-r，移植后MRD监测建议首选MLL-r，WT1基因可作为有益的参考指标。

## References

[1] Liu J, Wang Y, Xu LP (2014). Monitoring mixed lineage leukemia expression may help identify patients with mixed lineage leukemia--rearranged acute leukemia who are at high risk of relapse after allogeneic hematopoietic stem cell transplantation[J]. Biol Blood Marrow Transplant.

[2] Gao MG, Ruan GR, Chang YJ (2020). The predictive value of minimal residual disease when facing the inconsistent results detected by real-time quantitative PCR and flow cytometry in NPM1-mutated acute myeloid leukemia[J]. Ann Hematol.

[3] Zhao XS, Jin S, Zhu HH (2012). Wilms' tumor gene 1 expression: an independent acute leukemia prognostic indicator following allogeneic hematopoietic SCT[J]. Bone Marrow Transplant.

[4] Arber DA, Orazi A, Hasserjian R (2016). The 2016 revision to the World Health Organization classification of myeloid neoplasms and acute leukemia[J]. Blood.

[5] 王 欣玉, 常 英军, 刘 艳荣 (2021). 多参数流式细胞术与实时定量PCR技术检测Ph阳性急性B淋巴细胞白血病异基因造血干细胞移植前微小残留病的预后意义比较[J]. 中华血液学杂志.

[6] Liu J, Zhang XH, Xu LP (2021). Minimal residual disease monitoring and preemptive immunotherapies for frequent 11q23 rearranged acute leukemia after allogeneic hematopoietic stem cell transplantation[J]. Ann Hematol.

[7] 秦 亚溱, 阮 国瑞, 李 金兰 (2005). 定量检测WT1基因表达水平在急性髓系白血病微量残留病监测中的意义[J]. 中华血液学杂志.

[8] Qin Y, Zhu H, Jiang B (2009). Expression patterns of WT1 and PRAME in acute myeloid leukemia patients and their usefulness for monitoring minimal residual disease[J]. Leuk Res.

[9] Wang Y, Liu QF, Xu LP (2015). Haploidentical vs identical-sibling transplant for AML in remission: a multicenter, prospective study[J]. Blood.

[10] 赵 晓甦, 秦 亚溱, 张 艳玲 (2015). 急性淋巴细胞白血病患者造血干细胞移植后监测E2A-PBX1融合基因的意义初探[J]. 中华血液学杂志.

[11] Döhner H, Estey E, Grimwade D (2017). Diagnosis and management of AML in adults: 2017 ELN recommendations from an international expert panel[J]. Blood.

[12] Zhao XS, Yan CH, Liu DH (2013). Combined use of WT1 and flow cytometry monitoring can promote sensitivity of predicting relapse after allogeneic HSCT without affecting specificity[J]. Ann Hematol.

[13] Deng DX, Wen JJ, Cheng YF (2021). Wilms' tumor gene 1 is an independent prognostic factor for pediatric acute myeloid leukemia following allogeneic hematopoietic stem cell transplantation[J]. BMC Cancer.

